# Determining the Repertoire of Immunodominant Proteins via Whole-Genome Amplification of Intracellular Pathogens

**DOI:** 10.1371/journal.pone.0036456

**Published:** 2012-04-30

**Authors:** Michael J. Dark, Anna M. Lundgren, Anthony F. Barbet

**Affiliations:** 1 Department of Infectious Diseases and Pathology, College of Veterinary Medicine, University of Florida, Gainesville, Florida, United States of America; 2 Emerging Pathogens Institute, University of Florida, Gainesville, Florida, United States of America; Kansas State University, United States of America

## Abstract

Culturing many obligate intracellular bacteria is difficult or impossible. However, these organisms have numerous adaptations allowing for infection persistence and immune system evasion, making them some of the most interesting to study. Recent advancements in genome sequencing, pyrosequencing and Phi29 amplification, have allowed for examination of whole-genome sequences of intracellular bacteria without culture. We have applied both techniques to the model obligate intracellular pathogen *Anaplasma marginale* and the human pathogen *Anaplasma phagocytophilum*, in order to examine the ability of phi29 amplification to determine the sequence of genes allowing for immune system evasion and long-term persistence in the host. When compared to traditional pyrosequencing, phi29-mediated genome amplification had similar genome coverage, with no additional gaps in coverage. Additionally, all *msp2* functional pseudogenes from two strains of *A. marginale* were detected and extracted from the phi29-amplified genomes, highlighting its utility in determining the full complement of genes involved in immune evasion.

## Introduction

Tickborne illnesses have become increasingly important as causes of disease in the United States in the last several years [1]. In particular, the incidence of human anaplasmosis, caused by *Anaplasma phagocytophilum*, has been increasing, hospitalizing 36% of patients infected and killing 0.6% [2]. The closely related organism *Anaplasma marginale* is a growing problem for the cattle industry. Despite being recognized for over 100 years, the production losses due to *A. marignale* continue to grow substantially [3]. Additionally, the true prevalence of *A. marginale* is unknown, as many countries that have endemic *A. marginale* infection do not monitor infection rates; despite this, *A. marginale* is the 28^th^ most common cause of lost production among all OIE reportable diseases.

Control of these diseases is made difficult by their mechanisms for evading the immune system. This has been best studied in *A. marginale*, which expresses variants of the major surface proteins *msp2* and *msp3* [4]. This creates a wide repertoire of expression site variants through segmental gene conversion [5], [6], [7], which increases in complexity over time [8], facilitating immune system evasion. While this generally prevents additional strains from infecting animals already infected with another strain, strains with a unique functional pseudogene are able to generate novel variants, allowing superinfection [9].

However, determining the number and composition of these pseudogenes has proven extremely difficult. Due to the nature of the functional pseudogenes, the previous research into pseudogene repertoires has used Southern blotting, gel extraction, cloning, and sequencing [10], a laborious process that has hindered analysis of the pseudogene repertoires of multiple strains. While next-generation sequencing has made investigation of new strains less labor-intensive, it requires substantial amounts of DNA, and experimental infection of animals and is not suitable for examination of large numbers of strains [11].

## Methods

### DNA isolation

DNA samples from *A. marginale* genomic DNA were isolated from bovine erythrocytes in a previous experiment [12]. *A. phagocytophilum* strain HZ DNA was isolated from organisms cultured in the infected promyelocytic leukemia cell line HL-60 (ATCC catalog number CCL-240). Host cell free organisms were prepared by needle aspiration and passage through 2.0 µm glass fiber filters as described [13].

### Phi29 amplification

10 ng aliquots of isolated genomic DNA were amplified with Phi29 DNA polymerase using the GenomiPhi V2 DNA amplification kit (GE Healthcare). Following amplification, aliquots were pooled and DNA purified by adsorption to silica-gel particles and elution in 10 mM tris-HCL, pH 8.5 (5 Prime Manual GelElute Extraction Kit).

### Genome sequencing

Samples of amplified and nonamplified genomic DNA from each strain were quantified on a Qubit fluorometer. From 5 to 20 µg amplified or nonamplified genomic DNA from each sample was provided to the Interdisciplinary Center for Biotechnology Research (ICBR) core facilities, University of Florida for library construction and sequencing on the Roche/454 Genome Sequencer according to standard manufacturer protocols. The SFF format flow files were returned by ICBR for bioinformatics analyses. All SFF files used in this experiment have been submitted to the Sequence Read Archive at NCBI under accession number SRA050330.2.

### Bioinformatics

The Mosaik [14] suite v.1.0.1388 was used to assemble reads to the corresponding reference genomes (CP000030 for *A. marginale* St. Maries [15], CP001079 for *A. marginale* Florida [11], and CP000235 for *A. phagocytophilum* HZ [16]). MosaikCoverage was used to graph genome coverage. To compare coverages over specific pseudogenes BAM format files from Mosaik alignments were viewed in Artemis, as described previously [12]. In addition, all sequences were assembled *de novo* using Newbler v2.3, with the derived assembly values. Newbler output was also used for chimera detection; reads marked as potential chimeras were examined using a custom Perl program and BLAST [17] to eliminate matches where the read coordinates were separated or overlapped by more than 20 nucleotides. Single nucleotide polymorphisms (SNPs) were detected using CLC Genomics Workbench v.4.6.1, comparing each genome to its associated reference genome.

MSP2 functional pseudogenes of *A. marginale* were extracted from sequenced reads of both amplified and nonamplified DNA as follows. First, if necessary, barcodes were removed with the cutadapt tool (Marcel Martin, http://code.google.com/p/cutadapt/), reads were then filtered on Galaxy (main.g2.bx.psu.edu) to extract all reads longer than 400 bp; these reads were screened for the presence of a 146 bp segment of the 5′ conserved region present in all msp2 pseudogenes but not present in the related msp3 family (TTAAGGGAGGTAAGAAGTCTAATGAGGATACAGCCTCAGTATTCTTATTAGGAAAGGAGTTAGCATATGATACAGCAAGAGGTCAGGTAGACCGTCTTGCCACTGCTTTAGGTAAGATGACTAAGGGTGAAGCTAAGAAGTGGGGT). Any reads that contained this sequence (allowing up to 5 mismatches) were identified with MosaikAligner using parameters -hs 11 –mmal –min 146 –mm 5. These reads were converted to fasta format using MosaikText, samtools and custom scripts and then aligned with Mafft. The aligned fasta files containing the conserved 5′ sequence were separated into groups of similar aligning sequences with Jalview. Any reads containing the region that projected into the hypervariable region were then compared and finally edited manually with Se-Al v.2.0a11 (Rambaut Research Group, University of Edinburgh, http://tree.bio.ed.ac.uk/software/seal/) to form the final amino acid and nucleotide sequence of each extracted group. The consensus sequence containing the conserved 5′ sequence, the hypervariable region and the conserved 3′ sequence (typically encoding LGKELAY to MANNIN) from each group was exported and compared to the reference sequences.

## Results

### Sequencing Statistics

**Figure 1:**
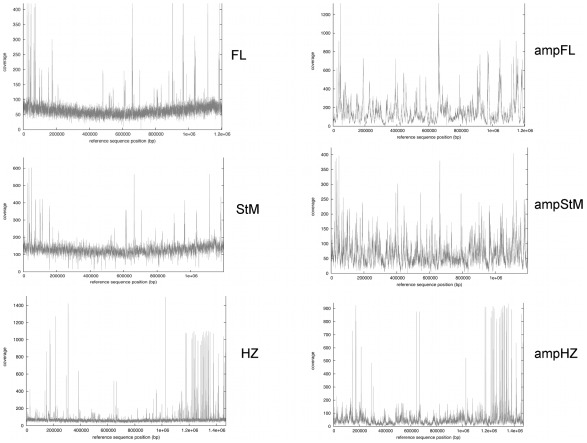
Genome coverage graphs of Florida (FL) and St. Maries, Idaho (StM) strains of *A. marginale* or the HZ strain of *A. phagocytophilum*. Sequencing reads derived from either amplified or nonamplified genomic DNA were aligned with the respective homologous reference genomes using Mosaik. Coverage was obtained across the entire genomes, although coverage levels were more variable with respect to genome location using amplified DNA.

The sequencing statistics for pyrosequencing of the three nonamplified and three amplified genomes are given in Table 1. For two of the three sets, the amplified genomes were mapped to fewer contigs than the nonamplified genomes. This is likely due to increased depth of coverage from the increased numbers of reads, allowing spanning of short repetitive areas. In general, amplified sequences had a smaller percentage of the total reads map to the genome (87.5% to 93.8%) compared to nonamplified sequences (90.3% to 99.1%). Genome coverage graphs are shown in Figure 1. The coverage of the amplified genomes was complete across most genome regions, albeit with significantly increased variability in coverage levels, similar to previous findings [18]. Areas of substantially increased coverage are similar between the amplified and nonamplified genomes, and tend to correlate with the location of functional pseudogene loci [11], [15]. These loci contain repetitive elements that lead to the spikes in coverage at those locations.

**Table 1:** Genomic sequencing statistics.

Original genome size	1,202,435		1,197,687		1,471,282	
	FL	FL amp	SM	SM amp	HZ	HZ amp
**Read Statistics**						
Total reads	35,670	468,695	63,260	278,066	88,420	272,709
Total mapped reads	35,233	455,532	62,271	273,539	79,133	257,105
Total bases	11,889,078	203,308,320	27,918,683	83,943,863	37,509,146	73,390,330
Total mapped bases	11,779,891	190,652,533	27,394,291	78,642,875	33,885,705	64,189,292
**Contig>500 bp Statistics**						
Number of contigs	65	20	31	24	56	61
N50 contig size	27,393	72,652	60,895	114,814	47,931	47,757
Average contig size	18,199	59,659	38,393	49,560	25,657	23,414
Largest contig	63,808	271,598	142,576	209,761	99,143	106,111
Number of bases	1,182,958	1,193,196	1,190,200	1,189,443	1,436,840	1,428,294
Q40+ bases	1,157,026	1,192,198	1,186,779	1,186,691	1,431,402	1,419,016

### Chimera Formation

One of the issues that has been raised with high-throughput sequencing is the development of chimeras, given the amplification step associated with pyrosequencing. Previous studies have found rates of chimera formation vary tremendously [19]; however, many of these are examining amplification of 16S rRNA genes, which may increase the rate of chimera formation because of the similarity of the targets being sequenced. Table 2 lists the rates of chimera formation for the six samples sequenced. The amplified genomes had generally higher chimera rates. All samples had chimera rates less than 4% of the total reads (1.61 to 3.37%). Interestingly, a majority of the chimeric sequences in the amplified genomes were from different strands. The majority of sequences from the nonamplified *A. marginale* genomes were generally from the same strand, while those from *A. phagocytophilum* were from opposite strands.

**Table 2:** Chimeric sequence statistics for amplified and nonamplified DNA.

		FL		FL amp		SM		SM amp		HZ		HZ amp	
Total Reads		35,670		468,695		63,260		278,067		88,421		272,710	
Total Chimeras		12	0.03%	7,526	1.61%	17	0.03%	5,136	1.85%	145	0.16%	9,194	3.37%
Chimeras	Same Strand	7	0.02%	573	0.12%	10	0.02%	369	0.13%	58	0.07%	1,134	0.42%
Chimeras	Different Strand	5	0.01%	6,953	1.48%	7	0.01%	4,767	1.71%	87	0.10%	8,060	2.96%

### Single Nucleotide Polymorphisms

Table 3 lists a comparison of SNPs between the amplified and nonamplified genomes. These numbers are slightly different from those previously published [12] because of different software to determine SNPs (CLC Genomics Workbench vs. Newbler). While the amplified genomes had slightly increased numbers of total SNPs, the SNP rate becomes similar when when SNPs are restricted to those occurring in all reads (100% frequency). Further, the numbers of transitions, transversions, synonymous, non-synonymous, and intergenic SNPs are all similar between amplified and nonamplified when SNPs are restricted to those with 100% frequency.

**Table 3:** Single nucleotide polymorphism statistics (freq – frequency, Syn – synonymous).

									100% frequency		
		Total SNPs	100% freq	% of total	Transitions - 100% freq		Transversions - 100% freq		Non-Syn	Syn	Intergenic
Florida	Normal	45	26	57.8%	11	42.3%	15	57.7%	11	12	3
	Amplified	73	22	30.1%	9	40.9%	13	59.1%	11	7	4
St. Maries	Normal	128	79	61.7%	32	40.5%	47	59.5%	48	15	16
	Amplified	136	83	61.0%	37	44.6%	46	55.4%	51	16	16
HZ	Normal	38	6	15.8%	3	50.0%	3	50.0%	4	2	0
	Amplified	67	7	10.4%	3	42.9%	4	57.1%	5	1	1

### MSP2 Pseudogene Detection

**Figure 2:**
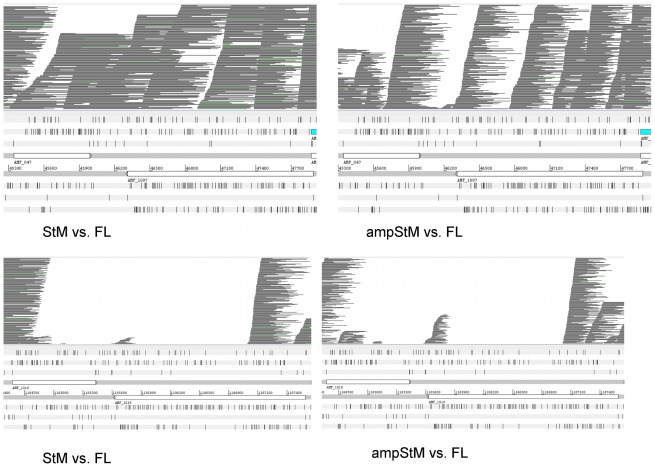
Detection of shared and different pseudogenes between strains of *A. marginale*. Top panel: Mosaik alignment of sequencing reads from the StM strain with the FL strain as reference; left is nonamplified StM genomic DNA, right is amplified St. Maries genomic DNA (the region of the FL strain encoding the *msp2/msp3* gene pair AMF_047/AMF_1097 is shown). Lower panel: alignment of reads over the *msp2/msp3* gene pair AMF_1018/AMF_1019. The lack of corresponding genes in the St. Maries strain is revealed by gaps in coverage.

*Msp2* functional pseudogenes may be similar or different between strains of *A. marginale* and this has been linked to the ability of strains to superinfect an already persistently infected animal. We showed previously that conservation of pseudogenes between strains could be rapidly determined by viewing pyrosequencing reads as BAM files aligned with reference genomes. Different pseudogenes appear as gaps in coverage when comparing pyrosequenced reads to the reference genome. We tested this with phi29 amplified sequences, with similar resultsto those described previously, for both Florida and St. Maries strains of *A. marginale*. *Msp2* and *msp3* pseudogenes with less than 92% identity between Florida and St. Maries were readily detected whether the BAM files were derived by alignment of amplified or nonamplified genome reads with the reference genome. Two examples are given in Figure 2. In the top panel, the *msp2/msp3* pseudogene pair AMF_047/AMF_1097 of the Florida strain is compared with pyrosequenced St. Maries. These pseudogenes are shared (100% identity) between the two strains and there are no gaps in coverage. In the lower panel the *msp2/msp3* pseudogene pair AMF_1018/AMF_1019 is compared between Florida and St. Maries strains. For these pseudogenes the closest match in St. Maries is 91% for AMF_1018 and 55% for AMF_1019. This is revealed by gaps in coverage, whether one uses amplified or nonamplified genome DNA from St. Maries.

**Figure 3:**
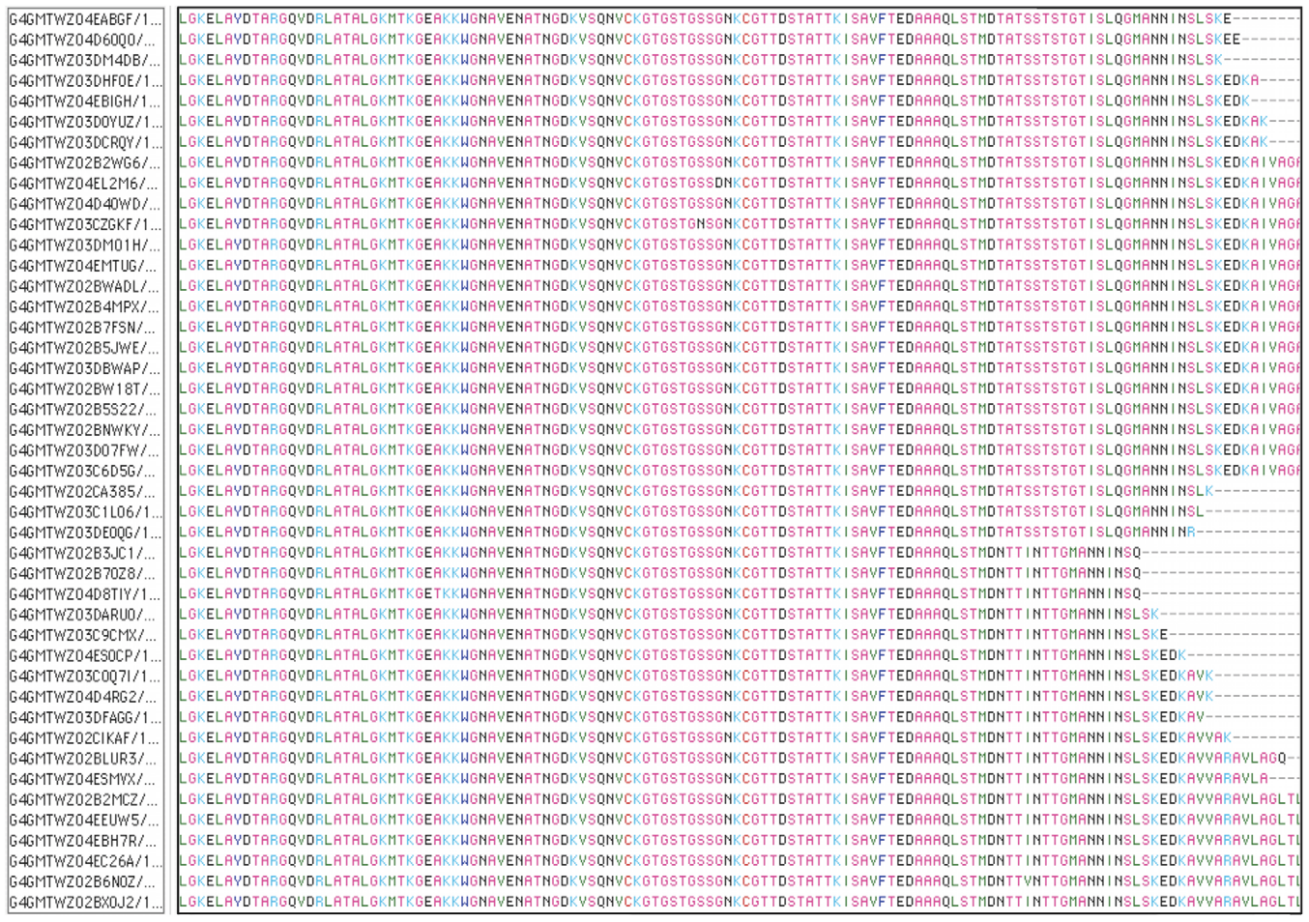
Extraction of different *msp2* pseudogenes from amplified genomic DNA using Se-Al. Following alignment of reads with Mafft and separation into similar sequence groups, the read groups were edited manually with Se-Al. There are clearly two major groups of sequence reads represented in this alignment, which are derived from pseudogenes AMF_872 and AMF_1018 of the FL strain.

**Figure 4:**
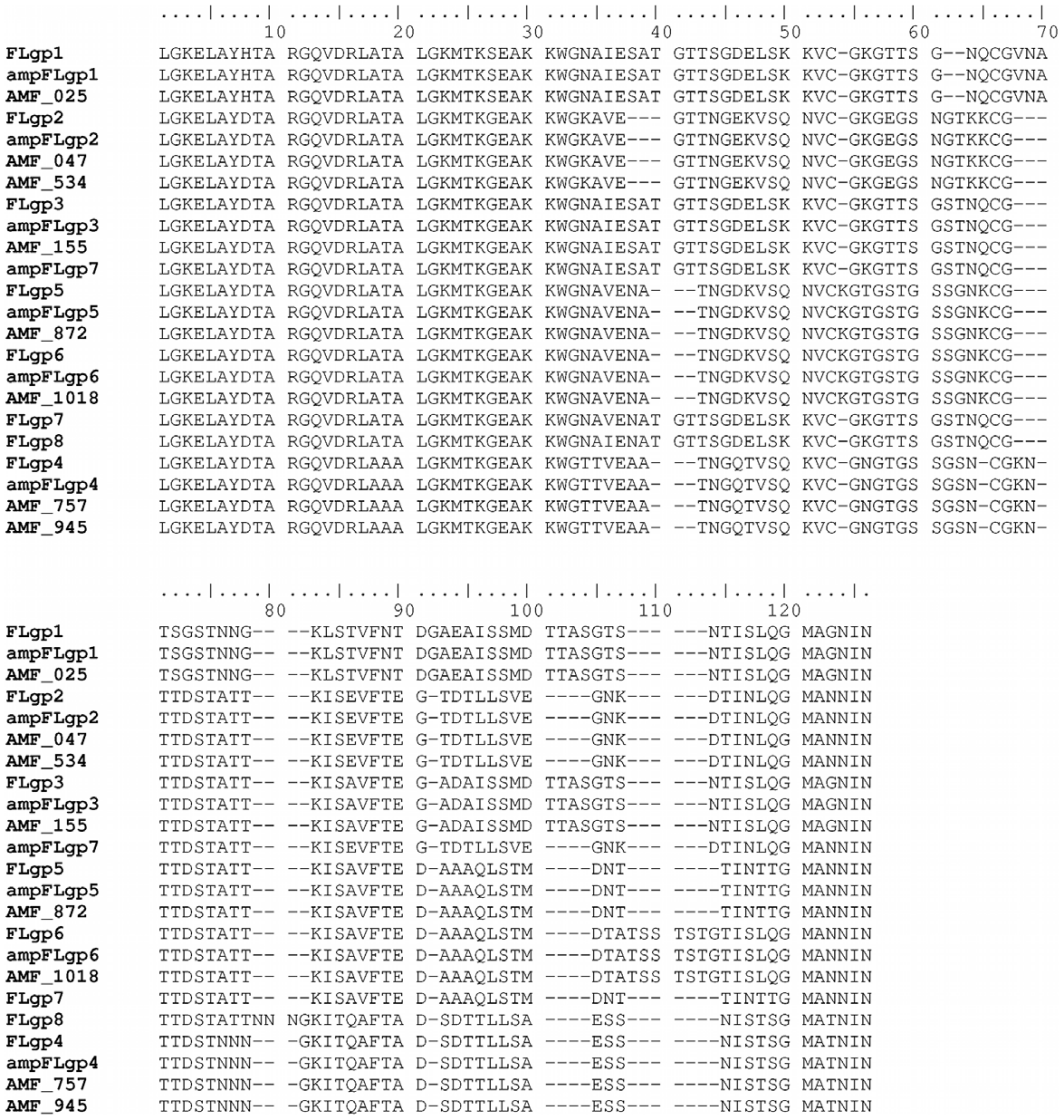
Alignment of all pseudogene sequences from amplified and nonamplified genomic DNA sequences extracted from the Florida strain with full-length pseudogenes from the previously sequenced Florida strain (CP001079).

**Figure 5:**
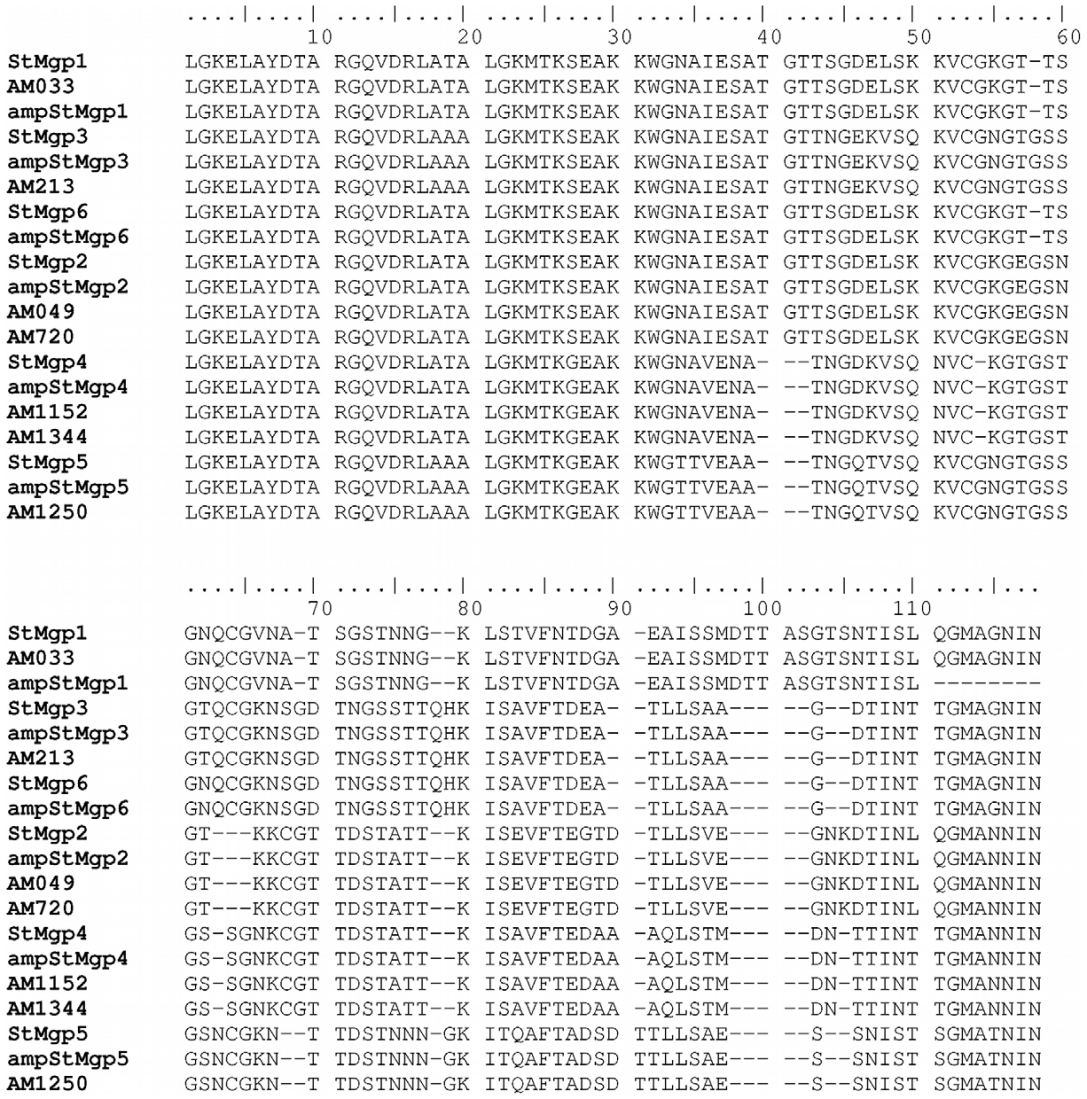
Alignment of all pseudogene sequences from amplified and nonamplified genomic DNA sequences extracted from the St. Maries strain with full-length pseudogenes from the previously sequenced St. Maries strain (CP000030).

It would be useful if, as well as revealing differences between pseudogene repertoires, the actual pseudogene sequences themselves could be extracted from a high-throughput read library. We considered that this might be possible using the 5′ conserved sequence flanking the hypervariable region as an alignment target in Mosaik. Accordingly, filtered read libraries (for length >400 bp) were aligned with the conserved 5′ sequence and those reads containing that sequence were extracted and aligned. An example of a final alignment of sequences from the amplified Florida genome using Se-Al is shown (Figure 3). From the alignment, it is evident that there are two groups of sequences. While some reads have individual sequencing errors, they clearly do not match the consensus sequences, which correspond to the known pseudogenes of the Florida strain AMF_872 and AMF_1018 (Figure 4). It is possible to differentiate these two pseudogenes in the amplified and sequenced genomic DNA although they are identical except at the extreme 3′ end. Accordingly, we then extracted all groups of msp2 pseudogene sequences from amplified and nonamplified genomic DNAs of Florida and St. Maries strains (Figures 4 and 5). All eight known pseudogenes in the Florida strain and all seven in the St. Maries strain were detected by pyrosequencing of amplified or nonamplified genomic DNAs. In addition, some sequences that did not match known pseudogenes, such as ampFLgp7, StMgp6, ampStMgp6, were detected. In several of these cases, these variants have been previously detected as msp2 expression site (ES) variants (ampFLgp7 = ES variant 198A; StMgp6 and ampStMgp6 = ES variant SGV1 [7], [20]), and are therefore considered authentic.

## Discussion

High-throughput sequencing techniques have yielded tremendous amounts of new information on pathogens, and have led to an explosion in the number of sequenced bacterial genomes [21]. However, many of these are organisms that have an established culture system, allowing isolation of large numbers of clonal organisms for DNA extraction. It will be valuable to be able to target individual genes and gene families from non-culturable organisms for structural analysis.

Here, we show that this technique is useful for examination of the intracellular *Rickettsiales* organisms *Anaplasma marginale* and *Anaplasma phagocytophilum*. Using as little as 10 ng genomic DNA, it is possible to obtain coverage across most genome regions. The sequence reads appear to be of similar quality from either amplified or nonamplified genomic DNA, with a higher number of chimeric sequences in amplified DNA. This strongly suggests that phi29 amplification creates an additional opportunity for chimera formation, leading to higher chimera rates. Despite the chimeras, it is possible to identify and compare gene differences between strains. The high numbers of non-chimeric reads allow for ready identification and elimination of chimeras, preventing interference with data analysis. It is unknown why non-amplified *A. phagocytophilum* had the majority of chimeras arise from different strands, when both *A. marginale* strains had the majority arise from the same strand; this may be the result of the increased numbers of repetitive sequences in *A. phagocytophilum*, which gives more opportunities for chimera formation.

Therefore, phi29 amplification, when coupled with proteomic approaches [22], will allow for better determination of vaccine targets conserved between all strains of these organisms, as current data shows a more distant evolutionary relationship of *A. marginale* subspecies *centrale* strains and much greater conservation of vaccine targets among *A. marginale* strains alone [12], [22], [23]. Further, this technique may be useful in examining populations of bacteria in vectors, without the necessity of culture. However, given the large number of bacteria present in many vectors [24], this may require large numbers of reads, as well as verification of a lack of specificity for a particular bacterium or group of bacteria.

Additionally, while genome assembly via pyrosequencing alone is not currently possible, given the nature and length of the repeats in these genomes, the increasing length of sequence reads from a variety of technologies will likely enable the closure of these genomes. Despite the lack of complete genome assembly, high-throughput sequencing allows for analysis of multigene families, detecting all of the functional pseudogenes in the sequences examined. These data should be valuable for many of the *Anaplasmataceae*, where the complement of functional pseudogenes has been linked to the ability of strains to cause superinfection and spread to new geographic locations. Such strain invasions disrupt pre-existing endemic stability and can cause disease outbreaks in herds naïve to these new strains. The ability to use amplified genomic DNA for such gene analysis opens the possibility of investigating organism population structure in carrier or persistently infected animals, which typically have low levels of circulating organisms, as well as sequencing whole genomes from outbreaks to determine the genetic diversity between outbreak strains and the interplay between endemic and outbreak organisms.

More broadly, in this study we analyzed the *msp2* gene family, having hypervariable regions that could be extracted and analyzed using filtered reads of length at least 400 bp, derived by pyrosequencing only 10 ng genomic DNA. The reads of interest were isolated from the total read pool by alignment with a short conserved sequence flanking the hypervariable region. As high-throughput sequencing achieves even longer read lengths, it will become possible to rapidly extract any specific gene and gene family that can be targeted using a known conserved sequence. This will enable determination of population structures for individual genes and should prove useful in vaccine development, epidemiologic analyses, and population responses to vaccine delivery or drug treatments.
